# Development of olanzapine solid dispersion by spray drying technique using screening design for solubility enhancement

**DOI:** 10.5599/admet.1998

**Published:** 2023-10-06

**Authors:** Leena Patil, Umakant Verma, Rahul Rajput, Pritam Patil, Aniruddha Chaterjee, Jitendra Naik

**Affiliations:** 1 University Institute of Chemical Technology, Kavayitri Bahinabai Chaudhari North Maharashtra University, Jalgaon-425001, Maharashtra, India; 2 Department of Chemical Engineering, Shri S'ad Vidya Mandal Institute of Technology, Bharuch, Gujarat, India; 3 Plastics Engineering Department, Plastindia International University, Vapi, Gujarat, India

**Keywords:** poorly soluble drug, Plackett–Burman design, surface morphology, drug-polymer interaction, encapsulation efficiency

## Abstract

**Introduction:**

Olanzapine (OLZ) is a psychotropic class drug commonly used to treat schizophrenia, bipolar disorder, and acute manic episodes. It has less water solubility, resulting in a slow dissolution rate and oral bioavailability. Therefore, the development in oral dosage forms is required to enhance the drug solubility.

**Method:**

The solid dispersion of olanzapine is prepared by spray drying technique. The solution of polyvinylpyrrolidone K-30 (PVP K-30), mono amino glycyrrhizinate pentahydrate (GLY), OLZ and silicon dioxide were dissolved in distilled water and ethanol and spray dried to get the solid dispersion. Solid dispersion was characterized for surface morphology, solubility, encapsulation efficiency (EE), X-ray diffraction (X-RD), Differential Scanning Calorimeter (DSC) and drug-polymer interaction by Fourier transforms infrared spectroscopy.

**Results:**

The amorphous nature of the drug's incorporation in solid dispersion was confirmed by X-RD analysis. Prepared solid dispersion showed higher solubility, 11.51 mg, than pure OLZ (0.983 mg ml^-1^), while the range of EE was found to be between 64 to 90 %.

**Conclusions:**

The solubility and dissolution rate of the OLZ can effectively increase by spray-dried solid dispersion. Plackett–Burman screening design plays a vital role in understanding the effect of independent variables on EE and solubility.

## Introduction

Among the potential drug candidates, 40 % of the drugs are poorly soluble in biological fluids; as a result, they have inadequate and uneven bioavailability. For this, development in oral dosage forms is required to enhance the drug solubility by bioavailability [1]. In addition, intestinal permeability and drug absorption rate are essential for drug development [2]. These drugs fall under the biopharmaceutical classification system (BCS) class II or IV [3]. Numerous techniques have been reported to enhance solubility for poorly water-soluble drugs. At the same time, many of these techniques have constraints regarding the micronized powder's particle agglomeration and poor flow feature [1]. However, solubility enhancement techniques commonly use pH adjustment, salt formation, self-emulsifying formulations, liposome and solid dispersion preparations, particle size reduction, complexation with cyclodextrin, *etc.* [4-6]. Solid dispersion is a widely used economic technique based upon the mechanisms of increasing surface area by reducing particle size, converting crystalline to the amorphous state, and drug wettability [7,8].

Physical instability is a significant challenge in the dispersion of OLZ; recent work has developed OLZ–polymer dispersions to understand the effect of the polymer on the drug recrystallization process and molecular mobility of the dispersion [9]. Several researchers reported polymeric nanocarriers in diverse delivery systems to improve the solubility of poorly soluble drugs. However, in the present study, PVP K30 polymer was used to develop stable solid dispersion of olanzapine owing to its exceptional absorbency and biocompatibility [10]. Generally, in pharmaceutical engineering, dispersion can be classified into three main categories: colloidal dispersion, molecular dispersion, and coarse dispersion, in which drugs can be dispersed or embedded in added molecules [11].

Olanzapine is one of the extensively used antipsychotic model drugs, commonly administrated to treat schizophrenia, bipolar disorder, and acute manic episodes [12]. OLZ has a significant drawback of poor water solubility, which gives uncertain absorption and inconsistent pharmacokinetic profile. Owing to these disadvantages, progress in the poorly water-soluble drug (OLZ) system for the efficient antipsychotic model is very important [13]. However, as per the reported literature, the drug solubility and dissolution rate of OLZ increase in amorphous form compared to the crystalline nature [12]. On the other hand, several foods, chemical, and pharmaceutical industries are more interested in employing drying techniques for their desired product. Amongst the various drying techniques, the spray drying technique is widely used in pharmaceutical industries for selected properties of dry drug particles [14]. In addition, the considerable significance of this technique is that it could be used for heat-sensitive and different solubility profile drugs. However, the operating parameters, inlet and outlet temperature, aspirator speed, and nozzle diameter play a fundamental role in the process optimization [15]. Optimizing operating parameters relating to feeding formulation and spray drying process is essential to elude difficulties like high moisture content and low yields in the product [16].

In the present research, stable solid dispersion of olanzapine was prepared by spray drying technique. The Plackett–Burman screening design was employed to investigate the effect of polyvinylpyrrolidone K-30 and mono amino glycyrrhizinate pentahydrate on encapsulation efficiency (EE) and solubility (mg ml^-1^) of olanzapine solid dispersion (OSD). The prepared spray-dried solid dispersion can effectively increase the solubility and dissolution rate of olanzapine.

## Experimental

### Materials

S. P. Pharmaceuticals, Jalgaon, India, generously gifted olanzapine. polyvinylpyrrolidone K-30 (PVP K-30) and Mono amino glycyrrhizinate pentahydrate (GLY) were kindly donated by Nanhang Industrial, P. R. China, and Sami Labs (Bangalore, India), respectively. Silicon dioxide amorphous was procured from Sigma-Aldrich (St. Louis, MO, USA). All other chemicals were of analytical grade and used as received.

### Method

#### Design of experiment

Plackett–Burman design (PBD) was employed using Design-Expert® Software (Stat-Ease Inc., Minneapolis, MN) to obtain the minimum number of experimental runs. PBD helps evaluate the independent variables' effect and identify the key ones influencing the responses, like solubility and encapsulation efficiency. The independent variables selected were the amount of GLY, mmol (*A*), concentration of PVP K -30, % w/v (*B*), nozzle diameter, mm (*C*), flow rate, ml min^-1^ (D), aspiration speed, rpm (*E*), inlet temperature, °C (*F*), solvent volume, ml (*G*) and solubility, mg mL^-1^ (*Y*_1_) and EE, % (*Y*_2_) were dependent variables. The framework of screening variables is presented in Table 1. The comparison of statistical parameters determined the significance of the PBD design. Two-dimensional (2D) contour plots and three-dimensional (3D) response plots were fabricated using Design-Expert® software (Version-8.0.7.1, Stat-Ease Inc., Minneapolis, MN). The polynomial model presented by equation (1) correlates the dependent and independent variables [17-20].


(1)

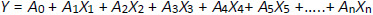



where *Y* is the response, *A*_0_ is the constant, and *A*_1_-*A*_n_ are the coefficients of the response values.

#### Preparation of olanzapine solid dispersion (OSD)

The spray drying technique prepared OSD with PVP K-30 and GLY. In brief, a known quantity of PVP K-30 and GLY were dissolved in distilled water and ethanol (1:3) to get a clear solution according to the design shown in Table 2. Afterward, OLZ was dispersed into the apparent solution of PVP K-30 and GLY with constant stirring of 30 minutes at room temperature. After that, Silicon dioxide (300 mg) as a glidant was added to the dispersion of OLZ. Lastly, spray drying (Spray Mate, Make JISL, and Mumbai) worked according to the parameters given in Table 2. The obtained product was kept in a desiccator over silica gel for further study [21-23].

### Physical characterization of solid dispersion

#### Saturation solubility studies

Higuchi and Connors method was employed to perform solubility studies of OLZ [24]. A surplus amount of OLZ was added into water containing various concentrations of GLY (mmol) and PVP K -30 (%). At first, prepared samples were sonicated for 5 min using a water bath sonicator (Leela Electronics, Leela Sonic 60) at room temperature. Then, shaking was performed for 48 h at 37±0.5°C using an incubator shaker (Remi Instrument, Mumbai). At equilibrium conditions, 5 ml aliquots were withdrawn and passed through a 0.22 μm syringe filter (PVDF). The OLZ content of each sample was analyzed by UV-vis spectrophotometer (U-2900, Hitachi, Japan) at 256 nm.

#### Encapsulation efficiency

The encapsulation efficiency of OLZ was determined by a UV-vis spectrophotometer [21]. A known quantity of OSD equivalent to 10 mg OLZ was mixed with 10 ml of ethanol and extracted in a phosphate buffer solution (pH 6.8). The answer was kept stirring for 30 min to evaporate ethanol, followed by filtration. Also, the residue was washed with a phosphate buffer solution. The EE was estimated in the filtrate after suitable dilution with phosphate buffer solution using a UV-vis spectrophotometer at 256 nm absorbance. The EE. of OLZ was determined using equation (2).


(2)

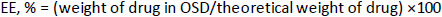



#### Surface morphology (FE-SEM)

The prepared OSD and OLZ surface morphology were examined by field emission scanning electron microscope (FE-SEM, S-4800, Type-II, Hitachi, Japan) at a working distance of 8.6 to 8.7 mm and accelerating voltage of 1.0 kV. For the morphological study, the sample was coated with gold to make them electrically conductive and mounted on metal stubs using double-sided adhesive tape under a vacuum [25].

#### Fourier transform infrared spectroscopy (FTIR)

Functional group analysis was studied for OLZ, OSD, GLY, and PVP K-30 and formulated by the KBr pellet method using an FTIR spectrophotometer (Shimadzu, FTIR-8400). The samples (2 mg) were mixed with KBr and compressed into a disc in a manual press. An FTIR spectrum was recorded in the wavelength region of 4000 to 400 cm^-1^ [21].

#### X-Ray diffraction analysis (XRD)

To verify the physical nature of the OSD, OLZ, GLY, and PVP K-30 is either crystalline or amorphous, the samples were examined by X-ray diffractometer (D8 advance, Bruker) with Cu Kα radiation (*λ* = 0.154060 nm). Cu anode at 40 kV monochromator voltage was performed for Data collection. The diffraction pattern was analyzed in area 20 < 2<<<<<<<<<< < 800 by a continuous scanning rate of 10° min^-1^ [26].

#### Differential scanning calorimetry (DCS)

The samples' thermal behaviour (melting point) was determined by using a Differential Scanning Calorimeter/TA-60 thermal analysis controller with an intracooler-2 cooling system (DSC-60, Shimadzu, Japan). Accurately weighed (3 to 5 mg) sample was placed in a standard aluminium pan and sealed carefully for scanning. The samples were scanned in the temperature range of 25 °C to 300 °C at 10 °C min^-1^ under a dry nitrogen atmosphere purge of 50 ml min^-1^ [27].

#### *In-vitro* drug release studies

An *in-vitro* drug release study was performed as per the USP monograph of olanzapine tablets in a wholly calibrated dissolution test apparatus (USP type II) assembled with an autosampler (Electrolab, Mumbai, India). Stirring was performed at the paddle speed of 50 rpm at 37±0.5 °C. Pure OLZ and OSD were positioned into a dissolution bowl, and a dissolution test was performed in 0.1 N HCl (pH 1.2) for one hour.

10 ml of aliquots from each bowl (2 ml for rinse and 8ml for analysis) were withdrawn at programmed time intervals (5, 10, 15, 20, 30, and 60 min.) and replenished with the contemporary fresh dissolution media to keep up the sink condition. Each sample was filtered through a 0.22 μ syringe filter, and the OLZ concentration was analyzed using a predetermined calibration curve at 256 nm [21].

## Results and discussion

### Statistical analysis of data

The PBD is a dominant and functional mathematical tool for identifying essential parameters on a response generated by conducting fewer trial runs; however, this design does not determine the exact quantity; it gives some necessary evidence about each parameter by performing relatively few experimental runs [18].

In the present work, the effect of independent variables on dependent variables was investigated at two levels: high level (+1) and low level (-1). The impact of 7 independent variables on the response (dependent variable) was studied by 12 experimental runs. Each level of the factor used in the experimental run is shown in Table 1. In contrast, the selection range of each factor was based on practical trial runs performed to investigate the effect of process parameters on response. The design matrix and experimental results in all the conditions are shown in Table 2. The impact of process variables upon response was modelled using the following polynomial equations,


(3)

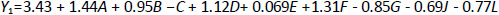




(4)

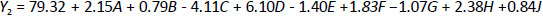



A positive value in the polynomial equations represents an increased response (synergistic effect) as the factor moves from a low to a high level. In contrast, a negative value indicates an inverse relationship (antagonistic effect) between the factors and response [21,28].

Response *Y*_1_, that solubility was influenced by factors like *A*, *B*, *C*, *D*, *F* and *G*, while J and L are the dummy factors shown in equation 3. On the other hand, equation 4 represents the significant influence of factors *A*, *C*, *D*, *F*, and *H* (dummy factor) on response *Y*_2_, that is, EE. Parameters *J* and *L* are dummy factors, which are the unknown factors that affect the EE. In contrast, factor *E* is the aspiration rate; water/ethanol loading was almost entirely evaporated at low rates. This decreased the probability of particle adhesion on the chamber walls and improved process yields [29].

Tables 3 and 4 represent the results of ANOVA, F-value, p-value, and mean square for the responses solubility and EE, respectively. The values of the correlation coefficient (*R*^2^) were found to be 0.9956 for the response *Y*_1_ (solubility) and 0.9968 for the response *Y*_2_ (EE), respectively. The Pareto chart was plotted to check the statistical significance of the PBD. It consists of the length of bars proportional to the absolute value of the estimated effects divided by the standard error, the *t*-value of the student's t-test. Among all seven factors, only *A*, *B*, *D*, *E* and *F* showed a significant positive effect on solubility, while the factors *C* and *G* negatively impacted solubility. From this, it was observed that the factors *C* and *G* are accountable for the high solubility of the OLZ, while in the case of EE, low levels of factors *C*, *E* and *G* are accountable for the elevated value of EE.

Diagnostics case statistics for various response variables are represented in Tables 5 and 6 with the actual value, predicted value and prediction error values of *Y*_1_ for all the experimental runs. The difference between actual and predicted values was very little; it concludes that the model was most suitable. In addition, the value of prediction error was meager.

### Solubility (Y_1_) and encapsulation efficiency (Y_2_)

**Figure 1.**
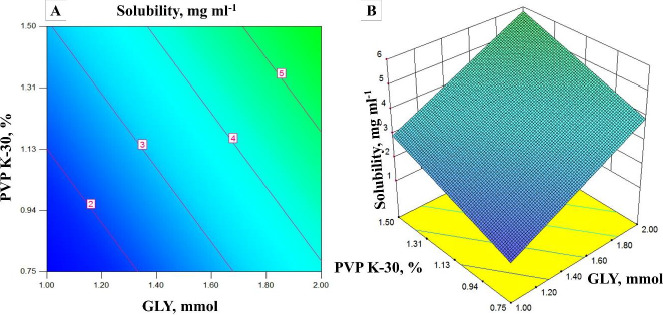
A) 2D surface plot showing the effect of independent variables on solubility and B) 3D surface plot showing the effect of independent variables on solubility

Different experimental runs of solubility and encapsulation efficiency are shown in Table 2. Effects of independent variables on each response were constructed in 3D response surface and 2D contour plots. Figure 1 (A and B) shows the impact of PVP K-30 and GLY on solubility. It could be predicted from the 2D contour plots and 3D response surface plots that an increase in the concentration of PVP K-30 and GLY increases the solubility of the OLZ in OSD. In contrast, the increase in the concentration of GLY decreases the EE, and the increase in the concentration of PVP K-30 increases the EE [30]. Vice versa, the relation between EE and GLY concentration could be due to the higher amount of GLY taking more time for precipitation. Range of OLZ solubility in OSD from 1.44 to 11.51 mg ml^-1^.

**Figure 2.**
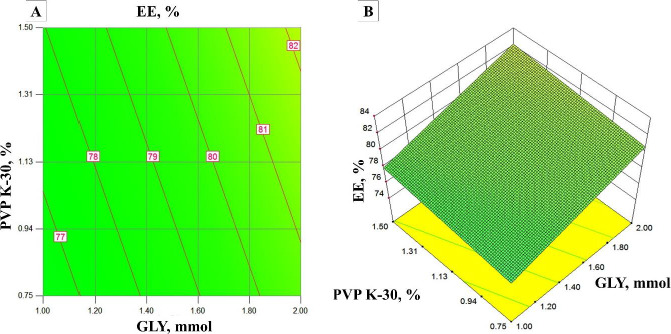
A) 2D surface plot showing the effect of independent variables on EE and B) 3D surface plot showing the effect of independent variables on EE

From the surface response plots and contour plots (Figure 2 A and B), it was observed that the EE increases with an increase in the concentration of PVPK-30 due to the high surface area of OSD. In addition, an increase in the concentration of GLY decreases the EE due to the leaching of the drug from OSD. EE was observed from 64 to 90 % of all experimental runs.

### Field emission scanning electron microscope (FE-SEM) analysis

**Figure 3:**
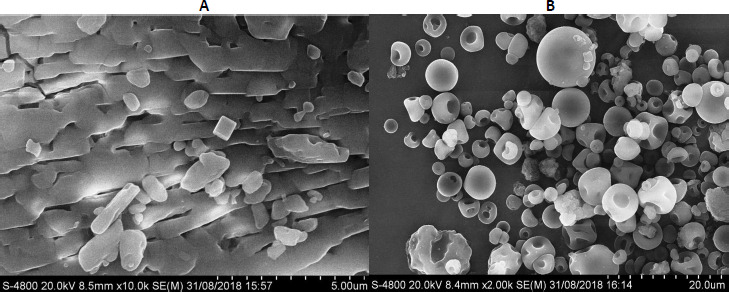
FE-SEM images of A) pure OLZ and B) FE-SEM image of OSD

Figure 3 shows the FESEM images of OLZ and OSD (Experimental Run 1, due to increased solubility as well as highest encapsulation efficiency). Clear crystal and rod-like morphology of the pure OLZ was observed in Figure 3A. Furthermore, the dent-like morphology of OSD was observed in Figure 3B, and early-stage shrinkage at a high drying rate probably happened. In addition, large-size agglomerated particles were observed due to the higher amount of surfactant. The effect of the polymer and surfactant's drying rate and viscoelastic properties was observed on the shape of smooth, indented, spherical surface morphology of OSD particles. As the concentration of surfactant increases, the atomized droplets drop from the hot air medium, and spherical shape particles are obtained. Therefore, it might be due to the highly prone interaction of solid dispersion with a solvent, which leads to faster dissolution as well as an increase in solubility [8,31].

### Fourier transforms infrared spectroscopy (FTIR)

**Figure 4.**
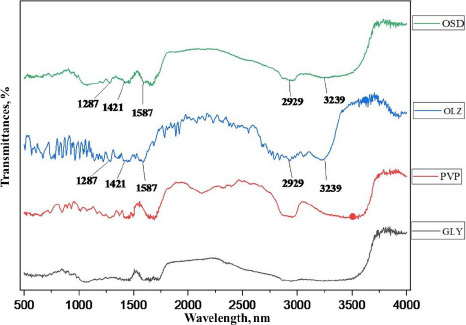
FTIR Spectra of OLZ, PVPK-30, GLY and OSD

FTIR spectrum of OSD, GLY, PVP K-30, and pure OLZ is shown in Figure 4. The FTIR spectrum of pure OLZ showed the principal peaks at 3239, 2929, 1587, 1421, and 1287 cm^–1^ corresponding to N-H and O-H stretching, C–H stretching, C=C stretching, C=N stretching, and C–N stretching, respectively. Pure OLZ characteristic peaks were present in the solid dispersion spectrum, which signifies no chemical interaction between the drug and carrier in the samples. In addition, a significant peak of pure OLZ at the absorption of 3239 cm^–1^ was observed in reduced form, with less sharpness and broader as the concentration of hydrophilic carrier was increased in the OSD, which might be due to physical interaction with excipients [32].

### X-ray diffraction (XRD)

**Figure 5.**
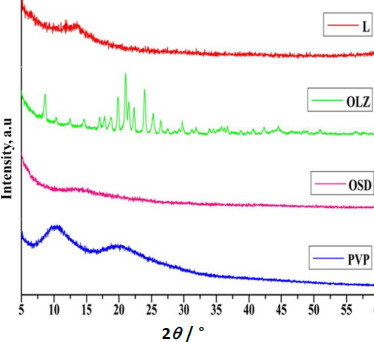
XRD images of PVP, OSD, OLZ and L (GLY)

XRD patterns of GLY, pure OLZ, OSD, and PVP K-30 are shown in Figure 5. Pure OLZ showed several distinct peaks, which confirmed the crystalline nature of the pure OLZ. There was no clear peak in the XRD pattern of GLY, and PVPK-30 indicates the amorphous nature. Furthermore, no distinct peaks were observed in OSD, indicating that the OLZ loaded into the PVPK-30 and GLY solid dispersion in an amorphous state [33,34]. In addition, the absence of crystalline peaks of pure OLZ in the diffractogram of OSD is mainly attributed to the hydrogen bonding of the amine- groups to the carboxyl groups of OLZ, leading to the disordering of the encapsulated OLZ [35,36]. Hence, the amorphous nature of the OSD reveals that most of the drug is distributed homogeneously within the PVP K-30 and GLY, which is responsible for high solubility and dissolution rate compared to pure OLZ.

### Differential scanning calorimeter (DSC)

**Figure 6.**
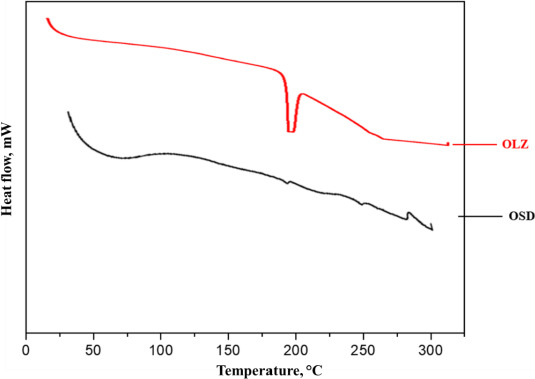
DSC thermogram of OLZ and OSD

Figure 6 shows the DSC thermograms of OLZ and OSD. The DSC thermogram of OLZ shows the intense endothermic peak at 193.37°C, which corresponds to the melting point of OLZ. The reduced intensity of the endothermic peak in OSD suggests that OLZ is likely trapped within OSD. The melting point of the OLZ in the OSD was shifted slightly due to the physical interaction of OLZ, GLY, and PVP K-30, which interrupted the rearrangement of the polymer chain due to intermolecular forces [27].

### *In-vitro* drug release study

**Figure 7.**
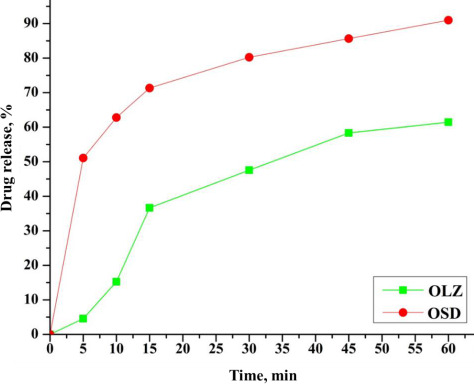
*In-vitro* drug release profile of OLZ and OSD (run 1)

The comparative *in-vitro* drug release profile of the OLZ and OSD (Run 1) is shown in Figure 7. 40 % of pure OLZ release was observed in 1 h.

In contrast, the dissolution rate of OLZ solid dispersion significantly increased. In addition, 90 % drug release of OSD was observed in 1 h. The dissolution rate of OLZ was increased due to the higher amount of hydrophilic carrier in solid dispersion. This is owing to the developed hydrophilic diffusion layer of PVP K-30 around the drug particles. Also, the wettability of the drug within the dissolution medium increases as the change is observed in hydrophobic nature. Factors like prevention of aggregation, amorphous nature, and agglomeration of the drug by carrier are accountable for the enhanced dissolution rate [5,36].

## Conclusions

In the present work, the spray drying technique successfully generated solid dispersion of OLZ using the Plackett–Burman design. From the results, it was observed that the encapsulation efficiency, solubility, and dissolution rate improved. The independent variables, such as PVP K-30 and GLY, significantly affect the response, viz., encapsulation efficiency and solubility. The prepared OSD particles show higher solubility compared to the pure OLZ. Also, the range of encapsulation efficiency was observed between 64 to 90%. XRD reveals that the amorphous nature of prepared particles enhanced the dissolution rate and solubility of OLZ. From the results, it could be concluded that the solubility and dissolution rate of the poorly soluble drug OLZ improves using PVP K-30.

## Figures and Tables

**Table 1.** Screening variables in a Plackett–Burman design.

Code	Independent variables	Level used actual (coded)	
		Low (-1)	High (+1)
*A*	Amount of GLY, mmol	1.00	2.00
*B*	Amount of PVP K-30, %	0.75	1.50
*C*	Spray Nozzle diameter, mm	0.30	0.50
*D*	Feed flow rate, ml min^-1^	10	15
*E*	Rate of aspirator, rpm	1200	1400
*F*	Drying chamber Inlet temperature, °C	110	120
*G*	Feed solvent, ml	90	120
*H*	Dummy factor 1*	-1	1
*J*	Dummy factor 2*	-1	1
*K*	Dummy factor 3*	-1	1
*L*	Dummy factor 4*	-1	1

*Unknown factor generated by software showing effect on EE and solubility of drug.

**Table 2.** The Plackett–Burman experimental design matrix and experimental results

Exp. run order	Independent variables											Dependent variables	
	*A* / mmol	*B* / %	*C* / mm	*D* / (ml min^-1^)	*E* / rpm	*F* / °C	*G* / ml	*H*	*J*	*K*	*L*	*Y*_1_ (solubility, mg ml^-1^)	*Y*_2_ (EE, %)
1	2.00	1.50	0.30	15	1400	120	90	-1	-1	1	-1	11.51	90.12
2	1.00	1.50	0.50	15	1200	110	90	1	-1	1	1	2.7	81.63
3	2.00	1.50	0.30	10	1200	120	90	1	1	-1	1	6.45	88.4
4	2.00	0.75	0.30	10	1400	110	120	1	-1	1	1	1.89	75.61
5	1.00	1.50	0.30	15	1400	110	120	1	1	-1	-1	3.18	87.41
6	2.00	0.75	0.50	15	1200	120	120	1	-1	-1	-1	6.02	86.71
7	1.00	1.50	0.50	10	1400	120	120	-1	-1	-1	1	1.03	64.52
8	1.00	0.75	0.50	10	1400	120	90	1	1	1	-1	1.49	70.45
9	1.00	0.75	0.30	10	1200	110	90	-1	-1	-1	-1	1.61	72.31
10	2.00	1.50	0.50	10	1200	110	120	-1	1	1	-1	1.44	68.56
11	2.00	0.75	0.50	15	1400	110	90	-1	1	-1	1	1.92	79.43
12	1.00	0.75	0.30	15	1200	120	120	-1	1	1	1	1.97	83.19

**Table 3.** Analysis of variance for solubility (*Y*_1_)

Source	Sum of squares	df*	Mean square	*F* value	*p*-value (0.05)*** (Prob > *F*)
Model	104.84	9	11.65	49.78	0.0198
*A*	24.77	1	24.77	105.86	0.0093
*B*	10.86	1	10.86	46.42	0.0209
*C*	12.04	1	12.04	51.45	0.0189
*D*	14.93	1	14.93	63.80	0.0153
*E*	0.057	1	0.057	0.24	0.6702
*F*	20.61	1	20.61	88.08	0.0112
*G*	8.61	1	8.61	36.79	0.0261
*J*	5.77	1	5.77	24.67	0.0382
*L*	7.18	1	7.18	30.68	0.0311
Residual**	0.47	2	0.23	-	-
Corrected total	105.31	11	-	-	-

*Degree of freedom

**Standard deviation of the residual = 0.48

***Level of significance; Correlation coefficient = 0.9956

**Table 4.** Analysis of variance for encapsulation efficiency (*Y*_2_)

Source	Sum of squares	df*	Mean square	*F* value	*p*-value (0.05)*** (Prob > *F*)
Model	852.75	9	94.75	68.83	0.0144
*A*	55.47	1	55.47	40.30	0.0239
*B*	7.39	1	7.39	5.37	0.1464
*C*	202.21	1	202.21	146.90	0.0067
*D*	433.92	1	433.92	315.22	0.0032
*E*	23.46	1	23.46	17.05	0.0540
*F*	40.19	1	40.19	29.19	0.0326
*G*	13.70	1	13.70	9.95	0.0875
*H*	67.97	1	67.97	49.38	0.0197
*J*	8.43	1	8.43	6.13	0.1317
Residual**	2.75	2	1.38	-	-
Corrected Total	855.51	11	-	-	-

*Degree of freedom

**Standard deviation of the residual = 1.17

***Level of significance; Correlation coefficient = 0.9968

**Table 5.** Experimental and predicted values of the response, *Y*_1_

Run order	*Y*_1_ / mg ml^-1^			Prediction error, %*
	Actual value	Predicted value	Residual	
1	11.51	11.63	-0.12	-1.04
2	2.70	2.45	0.25	9.26
3	6.45	6.33	0.12	1.86
4	1.89	1.64	0.25	13.23
5	3.18	3.06	0.12	3.77
6	6.02	5.89	0.12	2.16
7	1.03	1.28	-0.25	-24.27
8	1.49	1.23	0.25	17.45
9	1.61	1.87	-0.25	-16.15
10	1.44	1.56	-0.12	-8.33
11	1.92	2.17	-0.25	-13.02
12	1.97	2.09	-0.12	-6.09

*Prediction error was calculated using the formula: [(Actual *Y*_1_ - Predicted *Y*_1_) / Actual *Y*_1_]×100

**Table 6.** Experimental and predicted values of the response *Y*_2_

Run order	*Y*_2_ / %			Prediction error, %*
	Actual value	Predicted value	Residual	
1	90.12	90.66	-0.54	-0.60
2	81.63	82.04	-0.41	-0.50
3	88.40	87.86	0.54	0.61
4	75.61	76.02	-0.41	-0.54
5	87.41	87.00	0.41	0.47
6	86.71	86.30	0.41	0.47
7	64.52	63.98	0.54	0.84
8	70.45	70.99	-0.54	-0.77
9	72.31	71.90	0.41	0.57
10	68.56	69.10	-0.54	-0.79
11	79.43	78.89	0.54	0.68
12	83.32	83.12	0.20	0.24

*Prediction error was calculated using the formula: [(Actual value - predicted value)/Actual value]×100
